# Multiple Sources of Contamination in Samples from Patients Reported to Have XMRV Infection

**DOI:** 10.1371/journal.pone.0030889

**Published:** 2012-02-20

**Authors:** Mary F. Kearney, Jonathan Spindler, Ann Wiegand, Wei Shao, Elizabeth M. Anderson, Frank Maldarelli, Francis W. Ruscetti, John W. Mellors, Steve H. Hughes, Stuart F. J. Le Grice, John M. Coffin

**Affiliations:** 1 HIV Drug Resistance Program, National Cancer Institute, Frederick, Maryland, United States of America; 2 Advanced Biomedical Computing Center, SAIC, Frederick, Maryland, United States of America; 3 Laboratory of Experimental Immunology, Cancer and Inflammation Program, National Cancer Institute, Frederick, Maryland, United States of America; 4 Department of Medicine, University of Pittsburgh, Pittsburgh, Pennsylvania, United States of America; 5 Department of Molecular Biology and Microbiology, Tufts University, Boston, Massachusetts, United States of America; University of Pittsburgh Center for Vaccine Research, United States of America

## Abstract

Xenotropic murine leukemia virus (MLV)-related retrovirus (XMRV) was reported to be associated with prostate cancer by Urisman, *et al.* in 2006 and chronic fatigue syndrome (CFS) by Lombardi, *et al.* in 2009. To investigate this association, we independently evaluated plasma samples from 4 patients with CFS reported by Lombardi, *et al.* to have XMRV infection and from 5 healthy controls reported to be XMRV uninfected. We also analyzed viral sequences obtained from supernatants of cell cultures found to contain XMRV after coculture with 9 clinical samples from 8 patients. A qPCR assay capable of distinguishing XMRV from endogenous MLVs showed that the viral sequences detected in the CFS patient plasma behaved like endogenous MLVs and not XMRV. Single-genome sequences (N = 89) from CFS patient plasma were indistinguishable from endogenous MLVs found in the mouse genome that are distinct from XMRV. By contrast, XMRV sequences were detected by qPCR in 2 of the 5 plasma samples from healthy controls (sequencing of the qPCR product confirmed XMRV not MLV). Single-genome sequences (N = 234) from the 9 culture supernatants reportedly positive for XMRV were indistinguishable from XMRV sequences obtained from 22R*v1* and XMRV-contaminated 293T cell-lines. These results indicate that MLV DNA detected in the plasma samples from CFS patients evaluated in this study was from contaminating mouse genomic DNA and that XMRV detected in plasma samples from healthy controls and in cultures of patient samples was due to cross-contamination with XMRV (virus or nucleic acid).

## Introduction

In 2006, a novel infectious agent, xenotropic MLV-related virus (XMRV), was identified by hybridization to an oligonucleotide chip (“virochip”) and reported to be associated with prostate cancer [1]. Subsequently, XMRV infection was reported to be associated with chronic fatigue syndrome (CFS) by Lombardi, *et al.* who detected XMRV, using PCR, in 67% of samples from CFS patients compared to 3.7% of samples from healthy controls [2]. Such high frequencies of XMRV infection prompted concerns about widespread XMRV infection and stimulated research to determine the prevalence of XMRV infection worldwide. These efforts failed to detect XMRV in patients with either prostate cancer or CFS, even among a subset of patients from the original Lombardi, *et al.* study [3], [4] [5]–[7] [8], [9] [10], [11]. These findings suggested that XMRV detection was the result of laboratory contamination [3], [12], [13], [14], [15], [16], which is possible whenever sensitive amplification methods are employed, such as PCR or viral replication in cell culture. False positive detection of XMRV in patient samples could arise from PCR amplification of contaminating XMRV nucleic acids or from amplification of closely related endogenous retroviruses in the mouse genome that are misidentified as XMRV. With regard to the latter possibility, several recent studies have shown frequent contamination of reagents and samples with mouse DNA [3], [14], [17]. In addition, strong evidence that XMRV detection was the result of laboratory contamination came from a recent report that XMRV originated as a recombinant virus between two endogenous MLV proviruses (PreXMRV-1 and PreXMRV-2) between 1993 and 1996 during passage of a human prostate cancer xenograft in nude mice [18]. Cells from the passaged xenograft gave rise to the 22R*v1* cell line, which produces large amounts of infectious XMRV and has been distributed to laboratories worldwide [19]. The recombination event that gave rise to XMRV required multiple crossovers, and it is extremely unlikely that this complex event could have occurred more than once. Consequently, any virus whose sequence is closely related to this exact recombinant virus (XMRV) must have arisen from laboratory contamination by XMRV or its descendants. Indeed, since the generation and distribution of the 22R*v1* cell line, sublines of several other human cell lines, including Jurkat, 293T, and LNCap, have been reported to be contaminated with XMRV or similar viruses in laboratories using the 22R*v1* cell line [12]. Other cell lines, also derived from cancers passaged in nude mice, have been shown to be infected with viruses derived from a variety of endogenous MLVs; however, these viruses are distinct from XMRV [15], [20], [21].

To investigate further the possibility that XMRV or MLV detection in patient samples was the result of laboratory contamination by XMRV or mouse DNA, we performed qPCR and single-genome sequencing analysis on plasma samples from CFS patients who were reportedly infected with XMRV [2] and from healthy, XMRV uninfected controls. We also performed single-genome sequencing on supernatants from cultures containing XMRV reportedly isolated from patient samples. Our analyses reveal strong evidence for three different types of laboratory contamination giving rise to false positive detection of XMRV in human samples: mouse genomic DNA contamination of plasma samples from CFS patients; XMRV nucleic acid contamination of plasma samples from healthy controls; and contamination with infectious XMRV in virus isolation cultures. These results indicate that detection of XMRV infection in the original study by Lombardi, *et al.* likely arose from laboratory contamination and cast serious doubt on claims of human infection by XMRV and other MLVs.

## Methods

### Patient Plasma Samples

Plasma samples from 4 patients with chronic fatigue syndrome (CFS) reported to be XMRV-infected by PCR and virus isolation, performed at the Whittemore-Peterson Institute (WPI) and the Leukocyte Biology Section (LBS), NCI-Frederick, respectively, and from 5 XMRV-uninfected, healthy controls were obtained from F. Ruscetti, NCI-Frederick with permission from J. Mikovits, WPI (Table 1). All donors signed informed consent forms and the study was approved by the Institutional Review Board at the WPI (Approval ID# IRB00000215). Blood samples were drawn into EDTA-containing tubes by Phlebotomy Services International at the donors homes on January 21, 2010 and shipped overnight to LBS. The blood was centrifuged in a BL2* laboratory used for tissue culture in the LBS, the plasma was removed, aliquoted, and frozen at −80. The specimens were not reopened prior to testing in the HIV Drug Resistance Program (DRP). Samples were blinded with respect to their putative XMRV status by WPI and LBS and were provided to the DRP in June 2010 for testing by our XMRV single copy assay (X-SCA) and our XMRV single-genome sequencing assay (X-SGS) described below. We assigned the 9 blinded plasma samples identification codes X1–X9. After completion of testing, samples were unblinded to compare our results with those obtained by WPI and reported by Lombardi, *et al.* (Table 1).

**Table 1:** Independent evaluation of plasma samples from patients with previously reported XMRV status.

DRP identifier (PID)	CFS status	XMRV status	X-SCA result (copies/ml plasma)^b^	X-SGS result	mouse COX2	mouse IAP
X1	negative	negative	negative (<56)	negative	negative	negative
X2^a^	CFS	positive	mouse (15113)	positive	positive	positive
X3^a^	CFS	positive	mouse (4730)	positive	positive	positive
X4	negative	negative	XMRV (177)	negative	negative	negative
X5^a^	CFS	positive	mouse (471)	positive	positive	positive
X6^a^	negative	negative	negative (<6)	negative	negative	negative
X7	negative	negative	XMRV (6423)	negative	negative	negative
X8^a^	CFS	positive	mouse (2689)	positive	positive	positive
X9	negative	negative	negative (<9)	NT^c^	NT	NT

a As reported in Lombardi, et al [2].

b As compared to an RNA standard (Figure 1).

c Not tested.

### Supernatants of Virus Cultures Positive for XMRV Isolation from Patient Samples

LNCaP cells were co-cultured by LBS with plasma, PBMCs or tissues (collected from bone marrow biopsies) from 9 clinical specimens collected from 8 patients with putative XMRV infection. Virus-positive supernatants were subsequently used to infect human foreskin fibroblasts (HFFs). The same samples were opened multiple times for subculture experiments. Experimental cultures were kept in separate incubators from positive control cultures but were used in the same biological safety cabinets at different times. Samples of the HFF supernatants were provided to the DRP in February 2011 and we assigned them identifying codes DRP ID 1–9 (Table 2). To evaluate viral sequences in these culture supernatants we extracted viral RNA, synthesized cDNA, diluted cDNAs ∼10^11 -^fold and performed X-SGS for *gag* and *env* as described below. The description of the patients' clinical symptoms, sample type used for virus isolation, and cell passaging details are shown in Table 2. An additional plasma sample was collected on March 11, 2010 from one of the 8 patients who had virus isolation performed. This plasma sample was among the 9 plasma samples described above that were tested by X-SCA (plasma identifier X5; culture supernatant identifier DRP PID 5). Single-genome sequences obtained from this plasma sample (X5) and the virus culture supernatant (DRP PID 5) were compared.

**Table 2:** Evaluation of cell culture supernatants from virus rescue experiments^a^.

DRP identifier	Clinical diagnosis	Patient specimen used for virus culture	Cells used to culture virus (passage)^b^
1	CFS	plasma	HFF (4)
2	CFS	B cells	patient B cells (10)
3^c^	lymphoma	PBMC	HFF (4)
4^c^	lymphoma	bone marrow	HFF (2)
5	CFS	plasma	HFF (8)
6	CFS	plasma	HFF (5)
7	prostate cancer	prostate tissue	LNCaP (unknown)
8	CFS	PBMC	HFF (8)
9	CFS	plasma	HFF (2)

a samples obtained from LBS.

b The indicated sources were inoculated onto LNCaP (8–12 passages) and used to infect HFF cells, which were then grown in culture for 2–10 passages, after which the supernatants were subjected to X-SGS.

c These samples were obtained from the same patient.

### Cell lines

DNA and supernatants from 22R*v1* cells and the mammary carcinoma TA3.Cyc-T1 cell line derived from strain A mice, obtained from V. KewalRamani, NCI, were analyzed by X-SCA and X-SGS. Results from these cell lines were used as controls for sequences obtained from patient plasma samples. A subline of 293T cells was obtained from the LBS for use as an uninfected control, but was determined by Western blot to be infected with MLV or XMRV, was confirmed to be XMRV by sequence analysis, and is referred to as 293T-XMRV. Culture supernatant and DNA from these cells were subjected to X-SGS for comparison to virus reported to be isolated from patient plasma and tissues. We handled all cell culture supernatants in an area designated for cell culture and not in clean areas designated for processing of patient samples.

### Nucleic Assay Detection with X-SCA

The X-SCA assay [22] is similar to the HIV single-copy assay (SCA) [23], and can be used to quantify XMRV nucleic acid in blood and blood products. In brief, virus from patient plasma is collected by centrifugation after addition of the Rous sarcoma virus vector RCAS, an internal virion control for recovery [21]. cDNA was prepared from RNA in the pellet using random DNA hexamers as primers and subjected to PCR amplification using primers that are well conserved among XMRV and endogenous MLVs. Consequently, efficient amplification is achieved from both templates, and the assay can be used to detect either XMRV or MLV sequences in patient samples. The Taqman probe used for detection of amplified products was designed to span the signature 9–24 nt deletion in the XMRV *gag* leader absent from all endogenous MLV sequences (with the exception of PreXMRV-2) [18]. This probe design results in a lower plateau level of fluorescence from sequences amplified from MLV than from XMRV (Figure 1a), likely due to inefficient binding and degradation of the probe during amplification of MLV templates (Figure 1a). The different fluorescence profiles produced by XMRV and MLV sequences permits their identification and differentiation [22].

**Figure 1:**
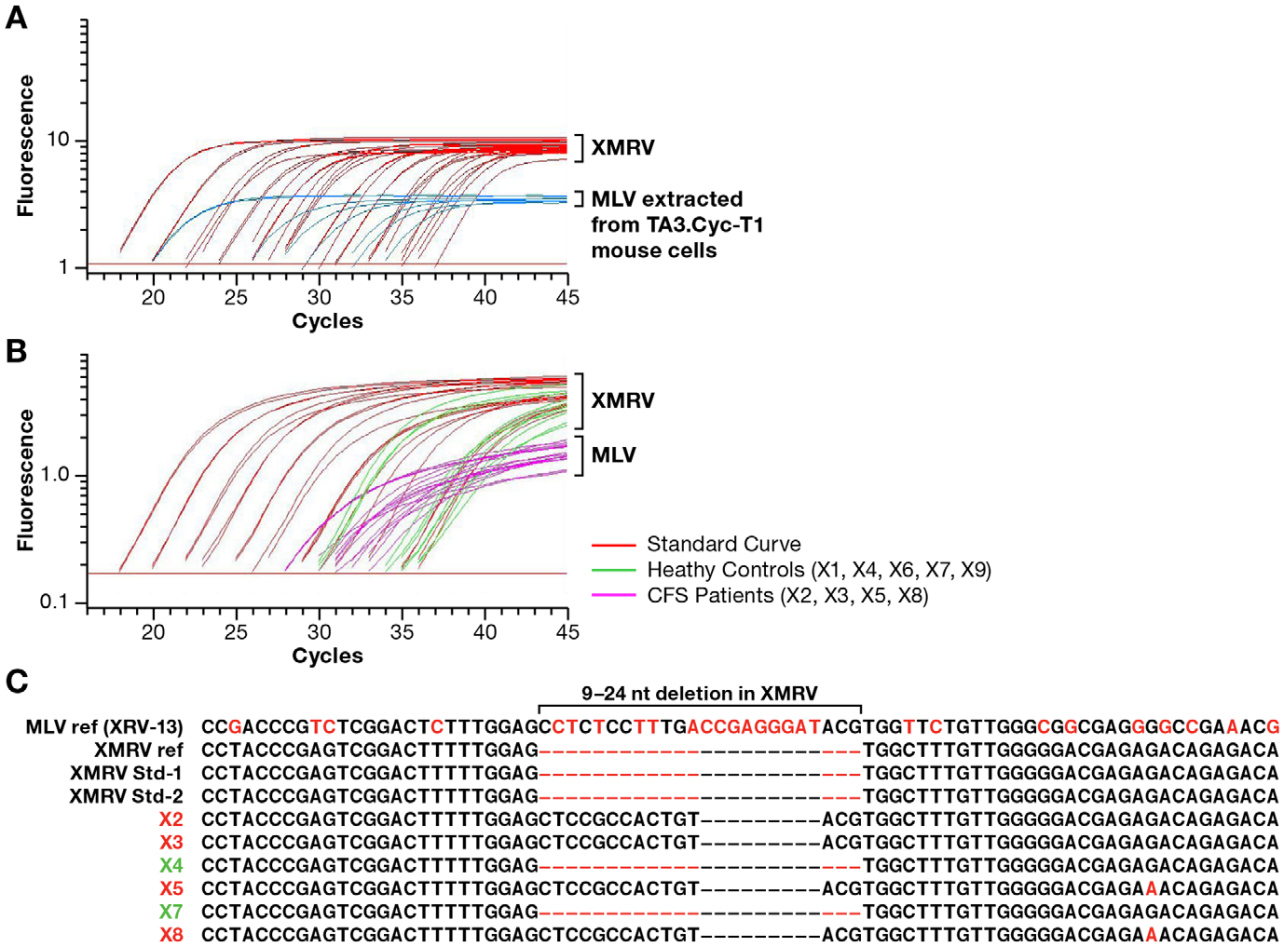
X-SCA amplification profiles of XMRV and MLV templates. Florescence intensity as a function of cycle number is shown for X-SCA amplifications initiated with dilutions of XMRV RNA and (a) endogenous MLVs found in mouse (TA3.Cyc-T1) genomic DNA or (b) patient plasma samples obtained from WPI and LBS. Red lines: XMRV standards; pink: CFS patient plasma samples; green: Normal control plasma. (c) Aligned sequences of the cloned amplicons detected in (b) from the indicated samples. The XMRV reference sequence matched vp62, virus from 22Rv1 cells, and Pre-XMRV-2.

### XMRV single-genome sequencing (X-SGS)

The HIV single-genome sequencing assay (SGS) [22] was modified for amplification and sequencing of single sequences derived from XMRV and MLV templates (X-SGS). RNA from virions pelleted from cell culture supernatant was used to synthesize cDNA using an oligo-dT primer with reaction conditions described in Supporting Information (Appendix S1). The cDNA was diluted to <1 copy per well and amplified using primers targeting XMRV and MLV sequences listed in Supporting Information (Appendix S2). cDNA synthesis and PCR reaction components and conditions were as reported previously [24]. Primers used to sequence single-genome amplicons are listed in Supporting Information (Appendix S2). The X-SGS *gag* protocol generates a 1.4 kb *gag* sequence and the X-SGS *env* protocol generates a 2.1 kb *env* sequence, which includes several of the distinctive recombination junctions described by Paprotka *et al.* in their paper on the origin of XMRV that demonstrated that XMRV is a laboratory artifact that arose by recombination between two MLVs called PreXMRV-1 and PreXMRV-2 [18]. The total product of each SGS PCR positive reaction was sequenced. Contigs were generated and sequences aligned using ClustalW (http://www.genome.jp/tools/clustalw) and MEGA5 (http://www.megasoftware.net). Alignment trees were constructed using a neighbor-Joining method in MEGA5.

### Detection of mouse mitochondrial DNA and mouse genomic DNA

We applied two previously developed assays to detect mouse DNA. The first was adapted from methods developed by W. Switzer, Centers for Disease Control and Prevention, Atlanta, GA, to measure the level of mouse mitochondrial DNA by detecting the cytochrome C oxidase subunit 2 (COX2) gene [25] (Appendix S3), and the second was adapted from O. Cingöz, Tufts University, Boston, MA, to detect intracisternal A particle (IAP) sequences [14]. Genomic DNA extracted from as few as 0.0034 cells from the mouse cell line, TA3.Cyc1, could be detected using the IAP primer set shown in Supporting Information (Appendix S4). Primer sequences and reaction conditions are described in Supporting Information (Appendix S3, S4).

## Results

### MLV-related sequences in plasma samples

X-SCA was performed on 9 plasma samples collected from donors, 5 of whom were reported in the 2009 Lombardi, *et al.* study [2]. Four of the 9 were CFS patients previously reported to be XMRV positive and 5 were healthy controls reported to XMRV negative [2] (Table 1). The samples were coded to blind the analysis with regard to the putative XMRV status of the donor. X-SCA detected high levels of MLV, but no XMRV, in all 4 samples from CFS patients (Figure 1). For reference, Figure 1a shows the differential fluorescence seen with the amplification of endogenous MLV (DNA extracted from mouse cells) and XMRV RNA standards. Figure 1b shows the fluorescence profiles of the sequences amplified from the CFS patient plasma samples compared to an XMRV RNA standard curve. These data show that the amplification profiles from the CFS patient samples mimic those of MLV and not XMRV. Cloning and sequencing of the X-SCA products confirmed that the amplicons did not contain the *gag* deletion specific to XMRV and preXMRV-2 sequences (Figure 1c). We also tested the samples for XMRV or MLV DNA by performing X-SCA without the reverse transcriptase (RT) enzyme and found that the CFS patient samples contained MLV DNA that was amplifiable (data not shown). These results show that the retroviral sequences detected in the CFS plasma samples were more similar to endogenous MLV DNA than XMRV RNA, suggesting high levels of contamination with mouse genomic DNA. X-SCA did not detect MLV sequences in the samples from the healthy control patients but did detect XMRV nucleic acid in two of the 5 samples (X4 and X7) reported to be healthy controls by WPI. The X-SCA product was sequenced and determined to be an exact match to XMRV (Figure 1c). Repeat X-SCA testing of both samples gave the same results, implying that the contamination occurred during preparation of the samples and not during performance of the X-SCA assay. We do not know the source of XMRV sequences in the two healthy control samples. Both samples gave a positive PCR signal in reactions lacking RT, but neither sample was positive for XMRV or MLV sequences by X-SGS using an oligodT primer (Table 1). The latter result suggests that the samples may have contained XMRV DNA but not viral RNA molecules. These findings, taken together with the fact that XMRV originated by recombination between two endogenous MLVs in a xenograft of prostate cancer passaged in nude mice, makes it likely that sample contamination with XMRV was from cloned or PCR-amplified XMRV DNA. Additional sample was not available to differentiate further between these two types of DNA contamination.

### Detection of mouse DNA sequences in patient plasma samples

**Figure 2:**
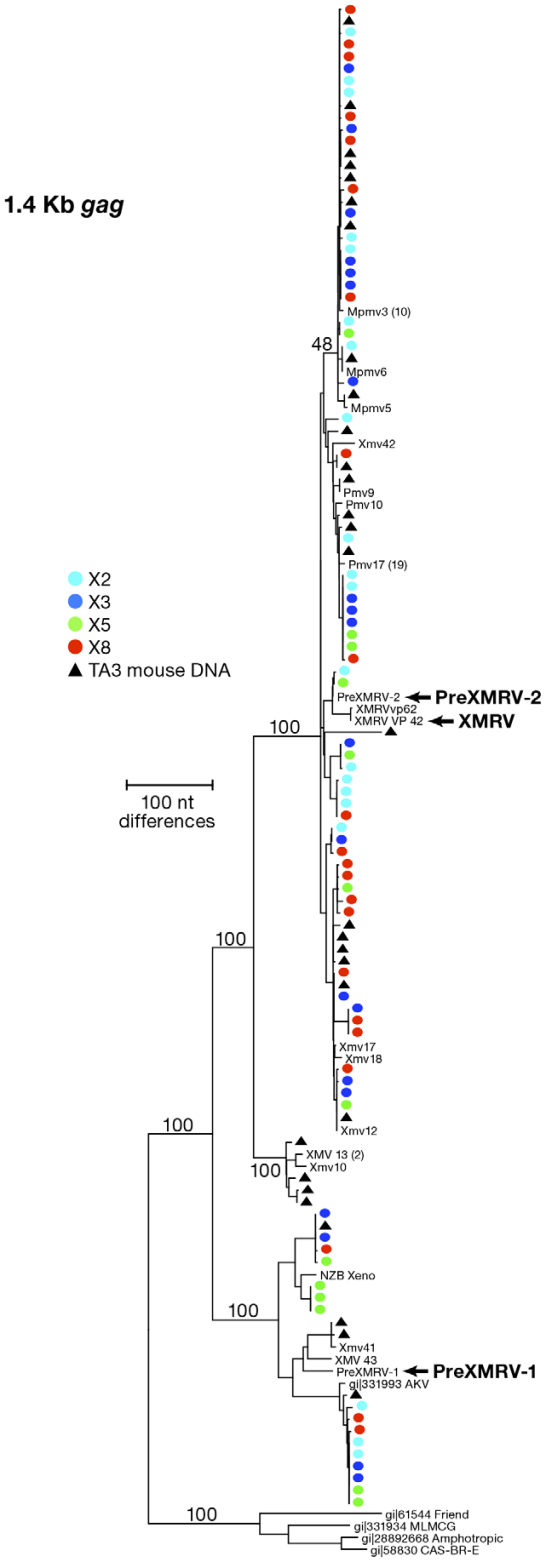
Neighbor-Joining Phylogenetic Tree of sequences obtained by X-SGS from CFS patients' plasma samples. Phylogenetic structure of gag single-genome sequences obtained from CFS patients X2 (aqua) X3 (blue) X5 (green), and X8 (red), and TA3.Cyc-T1 mouse cells (black triangles). Also includes sequences of endogenous MLVs extracted from the C57Bl6 genome sequence [26], as well as XMRV isolates are included for comparison. Where there are multiple identical sequences in the mouse genome, only one is shown, with the number of identical sequences in parentheses.

Because the X-SCA results suggested high levels of mouse DNA contamination in the CFS patient samples, we used three experimental approaches to detect mouse DNA. We tested for mouse mitochondrial DNA using an assay that detects the COX2 gene (Table 1), for mouse genomic DNA using an assay that detects intracisternal A-particle (IAP) sequences [14] (Table 1), and we performed X-SGS of a 1.4 kb fragment of the XMRV/MLV *gag* gene to determine the source of the amplified nucleic acid in plasma samples (Table 1, Figure 2). The three methods yielded concordant results for all 9 samples and provided unequivocal evidence that the plasma samples provided to us from 4 of the CFS patients originally reported [12] to be XMRV infected were contaminated with mouse DNA (Table 1). No mouse mitochondrial or genomic DNA was detected in the 5 samples from healthy controls by the COX2, IAP, or X-SGS assay.

**Figure 3:**
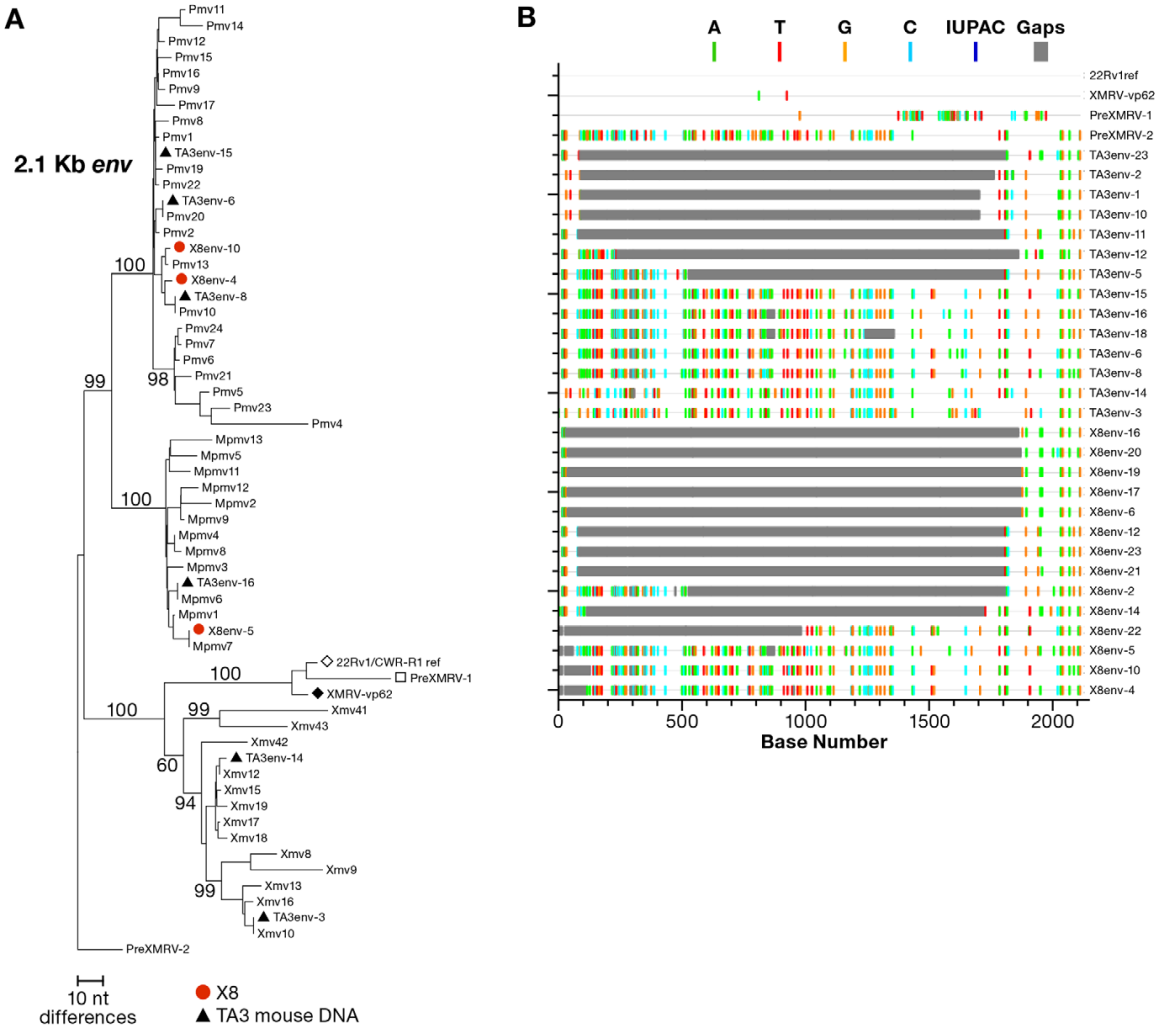
Env sequences obtained from plasma of patient X8. (a) Neighbor-joining analysis of full-length *env* sequences from patient X8, the full length TA3 sequences, and endogenous MLV as shown in Figure 2. (b) Highlighter plot of single-genome sequence alignment of *env* sequences from patient X8 and from TA3.CycT1 mouse cells.

To further analyze the MLV sequences obtained by X-SGS, we prepared neighbor-joining trees from the single-genome *gag* sequences obtained from the 4 CSF patient plasma samples and from sequences obtained from strain A mouse genomic DNA (extracted from TA3.Cyc-T1 cells). We compared these sequences to known endogenous MLV sequences [26] (Figure 2). Plasma samples from each of the 4 CFS patients contained MLV sequences indistinguishable from those found in mouse genomic DNA. Moreover, X-SGS did not detect XMRV sequences in any of the CFS patient samples, although, as expected for mouse DNA and described by Paprotka, et al [27], two of the sequences were an almost perfect match to preXMRV-2 in this region (one from X2 and one from X5). X-SGS was also performed on the *env* gene from patient X8 and compared to single-genome *env* sequences obtained from mouse cells (Figure 3). The majority of sequences in patient X8 contained large deletions, as seen in the highlighter plot of the alignment of Figure 3b (http://www.hiv.lanl.gov/content/sequence/HIGHLIGHT/highlighter_top.html). Similarly large *env* deletions were also present in the endogenous MLVs amplified by X-SGS from mouse genomic DNA, again confirming that mouse genomic DNA was the source of MLV sequences in the plasma samples from the CFS patients (Figure 3b).

### XMRV sequences of viruses obtained from co-cultures with patient samples

Although contaminating mouse genomic DNA can explain false positive results in PCR assays, it does not explain the isolation of replicating XMRV from patients reportedly infected with XMRV [2], [28], [29]. We therefore performed X-SGS analysis of supernatants from human foreskin fibroblasts (HFF) or LNCaP cells infected with virus reportedly isolated from plasma, PBMCs or tissue from 8 patients with putative XMRV infection (Table 2). One of these patients (Patient 5; Table 2) was among the 9 patients from whom we had evaluated viral sequences in plasma samples (patient X5 in Table 1). As described above, plasma from patient X5 was positive for MLV sequences but not XMRV sequences by X-SCA, and was contaminated with mouse DNA.

**Figure 4:**
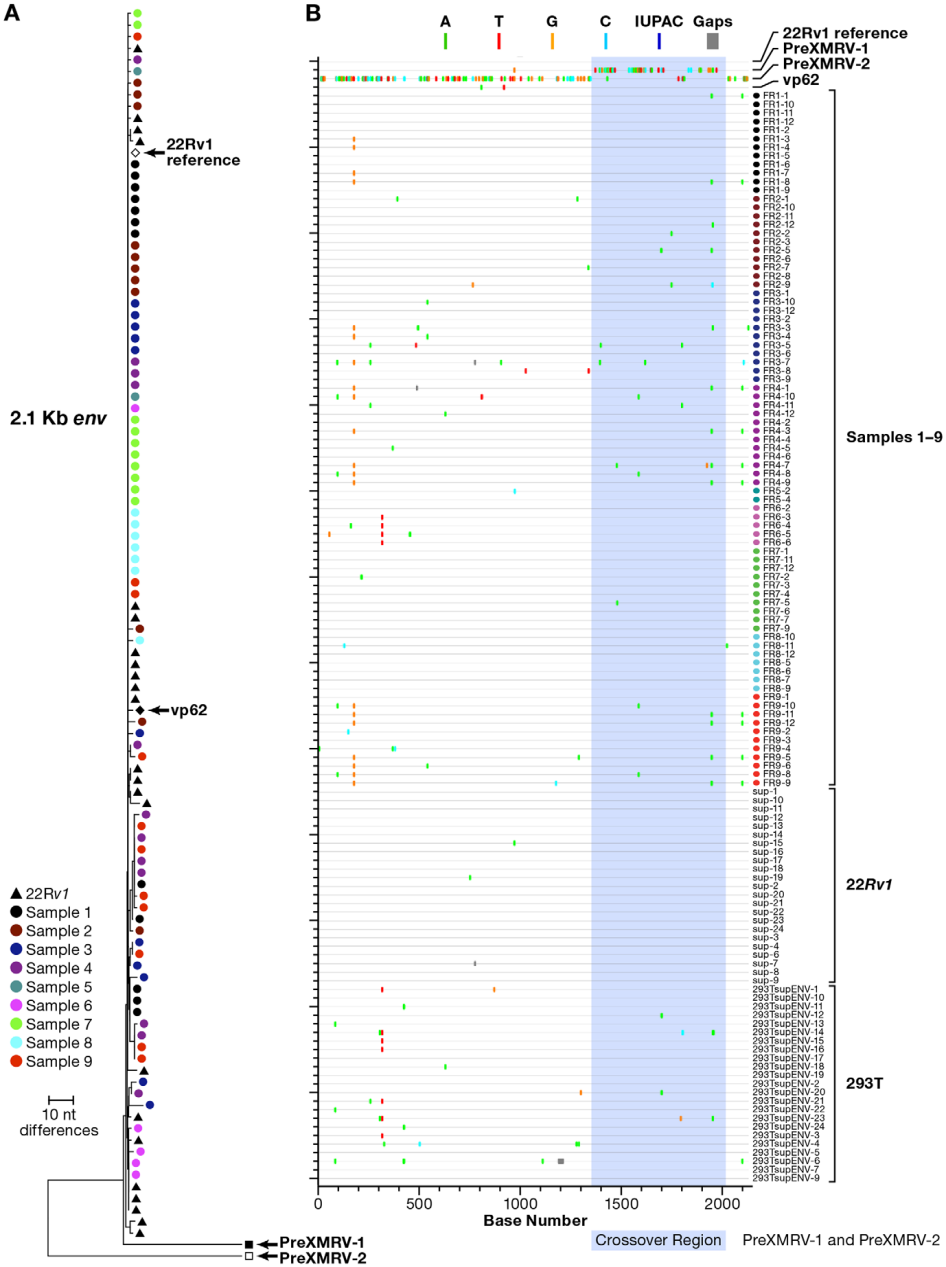
Env X-SGS of culture supernatants from virus isolations. (a) Neighbor-joining tree of single-genome *env* sequences from XMRV infected 293T cell supernatants, 22R*v1* cell supernatants, and supernatants from virus rescue experiments on samples 1–9. (b) Highlighter plots of the same sequences. PreXMRV-1 and -2 and the predicted recombinant (used as outgroup) [18] are included for comparison. The region of multiple crossovers inferred to have occurred between the two parental viruses is shaded.

**Figure 5:**
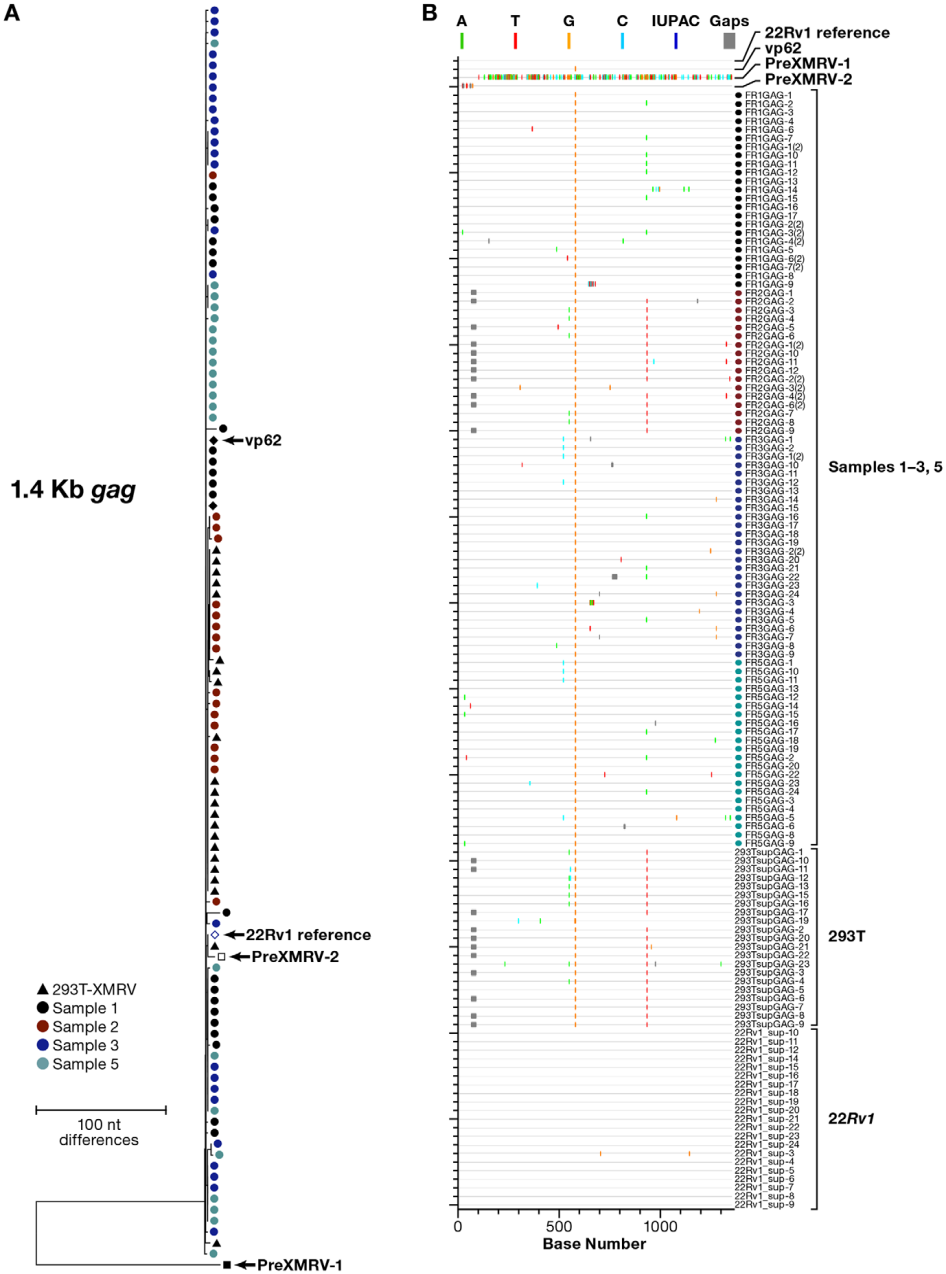
Gag X-SGS on culture supernatants from isolations. (a) Neighbor-joining tree of single-genome *gag* sequences from XMRV infected 293T cell supernatants, 22R*v1* cell supernatants, and supernatants from virus rescue experiments on patients 1–3, and 5. (b) Highlighter plots of the same sequences as compared to XMRV VP62. PreXMRV-1 and -2 and the predicted recombinant (used as outgroup) [18] are included for comparison.

X-SGS of *env* was performed on all 9 culture supernatants and on *gag* from a subset of three supernatants (PID 1, 2, 3). XMRV, but not MLV, sequences were detected in all 9 culture supernatants. As shown in Figures 4 and 5 and Table 3, all of the XMRV sequences detected in the supernatants were nearly indistinguishable from XMRV sequences found in well-characterized XMRV-infected cell lines. Specifically, Figure 4 shows the neighbor-joining tree and highlighter plot of XMRV *env* sequences found in the 9 culture supernatants compared to sequences obtained by X-SGS from 22R*v1* cells and from the 293T-XMRV subline. Phylogenetic analysis (Figure 4, 5) and calculations of genetic distance (Table 3) show that the viral sequences obtained from the co-culture supernatants from 8 different patients were nearly identical, and that these sequences are essentially the same as those from both 22R*v1* cells and 293T-XMRV cells. XMRV produced by 22R*v1* cells shows very low diversity (Figure 4, 5, Table 3), whereas the XMRV sequences from 293T-XMRV cells and co-culture supernatants were 2-16-fold more diverse (Table 3), consistent with acquisition of a few mutations during rounds of virus replication in cell culture that occurred during the co-culture procedure that included 2–10 passages. Moreover, the consensus sequences from the culture supernatants and the 293T-XMRV cells were identical to the consensus sequence of XMRV from 22R*v1* cells (with the exception of single nucleotide changes in PID 6 and 9). Because these analyses include a region in *env* (shaded in Fig. 4b) that contains multiple crossovers between PreXMRV-1 and -2 (events that occurred in the generation of XMRV and that are highly unlikely to occur twice as explained by Paprotka, et al. [18]), they provide very strong evidence that all of the viruses detected here arose from 22Rv1 cells.

**Table 3:** Pairwise diversity of XMRV *env* sequences from virus culture samples.

PID	% Intra-patient diversity^a^	% Distance from 22Rv1 consensus^a^
1	0.05	0.03
2	0.12	0.04
3	0.16	0.09
4	0.16	0.1
5	0.05	0.02
6	0.08	0.07
7	0.02	0.01
8	0.03	0.01
9	0.16	0.11
22Rv1 supernatant	0.01	0
293T supernatant	0.13	0.07

a average pairwise distance calculated in Mega 5.

X-SGS of *gag* was also performed using supernatants of cultures treated with samples obtained from PIDs 1–3 and 5. Although these sequences were very similar, more detailed analyses revealed that the culture supernatants from PIDs 1, 3, and 5 had *gag* sequences that were most closely related to XMRV from 22R*v1* cells, whereas the culture supernatant from PID 2 had sequences identical to those from 293T-XMRV cells (Figure 5).

PID 5 was the only patient for whom both plasma and virus culture supernatant were tested. We found sequences in the plasma sample that were derived from mouse genomic DNA, and were very different sequences detected in the co-culture supernatant, which were indistinguishable from the XMRV produced by 22R*v1* cells. These findings indicate that the plasma sample and the virus co-culture from this patient were contaminated from at least two independent sources, each of which led to different false positive results for XMRV infection.

## Discussion

Our analyses of plasma samples independently collected from CFS patients previously reported to be XMRV-infected [2], [29] and from healthy controls reported to be XMRV-uninfected [2], and of virus isolation co-culture supernatants identified three different types of sample contamination, leading to false positive detection of XMRV. First, we detected high levels of mouse genomic (both IAP and MLVs) and mouse mitochondrial DNA, but no XMRV sequences, in plasma samples from CFS patients, leading us to conclude that contaminating mouse genomic DNA in this set of plasma samples led to false positive PCR results for XMRV. Second, although the 5 plasma samples from healthy controls were free of mouse DNA, two of them contained contaminating XMRV nucleic acid – most likely plasmid or a PCR amplified DNA product – as shown by the PCR amplification profiles and confirmed by sequencing of the amplified product. Third, our analyses of sequences from viruses reportedly isolated from 8 patients with putative XMRV infection [29] revealed that the sequences did not differ among the patients and were indistinguishable from sequences of XMRV in XMRV-infected cell lines indicating that the cultures were cross-contaminated from infected cell lines used in the same laboratory. Specifically, the viruses reportedly isolated from patient samples exhibited very little diversity and were closely related to the 22R*v1* virus, consistent with a virus highly similar or identical to that found in the 22R*v1* cell line after a few cycles of virus replication in culture. These findings indicate that the putative isolations of replicating XMRV from patient samples were likely false positives as a result of cross-contamination of the cultures with XMRV from infected cell lines.

PCR and tissue culture are sensitive methods, and are, as a consequence, susceptible to contamination. Care must be taken both to prevent such contamination and to ensure that the analysis includes proper controls to exclude false positive results. The experimental samples and controls must be collected at the same time, using the identical materials, and processed together under identical conditions. Furthermore, it is important to develop strict criteria for declaring a sample positive, including a requirement that multiple methods should yield positive and negative results for the same samples, and the results should be reproducible. Our independent analyses of samples from patients reported to be XMRV-infected (12), refutes prior evidence of XMRV infection in these patients and argues against XMRV infection of human populations.

Our data show that there are at least three different ways contamination can be misinterpreted as XMRV or MLV infection of humans. The first is mouse DNA, which is a ubiquitous environmental contaminant that can find its way into experimental samples in many different ways. Examples include monoclonal antibodies or other bioproducts prepared in mice or mouse cells, chemicals, disposables, or other materials stored where mice can have access [3], [13], [14], [30], [31], [32], or handling of mouse specimens or cell lines in the same laboratory where human samples are being processed [12]. Inbred strains of mice contain around 60 MLV proviruses per haploid genome that can be detected by PCR with an *env-*specific probe [33], and some wild subspecies contain even more. Given that approximately 50% of the proviruses may be deleted in the *env* region (Fig. 3), one cell may contain over 200 proviruses that can be detected by PCR with *gag, pro, or pol* primers, increasing the potential that trace amounts of mouse DNA can give rise to a positive PCR signal. The second source of contamination is cloned or amplified XMRV DNA, including DNA being used as a positive control in diagnostic tests. A microgram of XMRV DNA is approximately 10^13^ copies. Any laboratory that works with either cloned or amplified XMRV DNA is a potential source of contamination. The third source of contamination is inadvertent spread of XMRV originating from 22R*v1* cells to indicator cells co-cultivated with clinical samples. Although this virus is quite sensitive to human restriction factors such as tetherin and APOBECs 3F and 3G, many established cell lines, like 293T, do not express these factors, and cross-contamination can occur even in laboratories with considerable virology experience, leading to subsequent spread to other cell lines, as was observed for the 293T-XMRV cells reported here. Inadvertent contamination of other human cell lines provides a plausible explanation for XMRV contamination even in laboratories that have never cultured the 22R*v1* cell line.

## Supporting Information

Appendix S1 **Protocol for synthesizing long cDNA fragments.**
(DOCX)

Appendix S2 **Primer sequences for XMRV PCR amplification and for sequencing.**
(DOCX)

Appendix S3 **Protocol for mouse COX2 Quantitative PCR.**
(DOCX)

Appendix S4 **IAP detection protocol and primer sequences.**
(DOCX)
