# Supplementary material for: Multiple Sources of Contamination in Samples from Patients Reported to Have XMRV Infection
Source: PLoS One. 2012 Feb 20;7(2):e30889. doi: 10.1371/journal.pone.0030889 (PMC3282701; doi:10.1371/journal.pone.0030889)
Supplement: Appendix S1 — Protocol for synthesizing long cDNA fragments. (DOCX) [file pone.0030889.s001.docx]

**Appendix S1**

*Protocol for synthesizing long cDNA fragments:*

50ºC for 1 hour (extension #1)

55ºC for 1 hour (extension #2)

70ºC for 15 min (inactivate enzyme)

4ºC for 5min

Add 2ul RNaseH (2U/ul)

37º for 20 min
