# Supplementary material for: Multiple Sources of Contamination in Samples from Patients Reported to Have XMRV Infection
Source: PLoS One. 2012 Feb 20;7(2):e30889. doi: 10.1371/journal.pone.0030889 (PMC3282701; doi:10.1371/journal.pone.0030889)
Supplement: Appendix S2 — Primer sequences for XMRV PCR amplification and for sequencing. (DOCX) [file pone.0030889.s002.docx]

**Appendix S2**

*X-SGS primer sequences*

*gag* PCR primers

XMRV 100+ TGG CCT CGC TGT TCC TTG GAG

XMRV 150+ AGC TCG GGG GTC TTT CAT TTG G

XMRV 1650- GAG GAC TAG GTG GTT CCT ACC T

XMRV 1700- CTG CCC GCG TTT TGG AGA CC

*gag* sequencing primers

XMRV 150+ AGC TCG GGG GTC TTT CAT TTG G

XMRV 1650- GAG GAC TAG GTG GTT CCT ACC T

XMRV 900+ GGA CAC CCG GAT CAG GTC CC

XMRV 950- GGT TTG ACC CAC GGA GGG GG

*env* PCR primers

XMRV 5700+ GAA CCT CGC TGG AAA GGA CC

XMRV 5750+ ACC GCT CTC AAA GTA GAC GGC AT

XMRV 8250- CTG AGG ACC ATC TGT TCT TGG CC

XMRV 8350- GAG AAG CGA GCT GAT TGG TTA GTT TAA AT

*env* sequencing primers

XMRV 5750+ ACC GCT CTC AAA GTA GAC GGC AT

XMRV 8250- CTG AGG ACC ATC TGT TCT TGG CC

XMRV 6600+ ATG TAG GGC CCC GCG TCC C

XMRV 6900- GAT ACT AGA CAC AGC CAG CAC TCT TG

XMRV 7700- TTA AAC AGT CCC TCA AAC CAC CCT TG

XMRV 7350+ TGT TGG GAG GAC TTA CTA TGG GCG
