# Supplementary material for: Multiple Sources of Contamination in Samples from Patients Reported to Have XMRV Infection
Source: PLoS One. 2012 Feb 20;7(2):e30889. doi: 10.1371/journal.pone.0030889 (PMC3282701; doi:10.1371/journal.pone.0030889)
Supplement: Appendix S3 — Protocol for mouse COX2 Quantitative PCR. (DOCX) [file pone.0030889.s003.docx]

**Appendix S3**

*Mouse COX2 qPCR*

MCox2-F2: TTCTACCAGCTGTAATCCTTA

MCox2-R1: GTTTTAGGTCGTTTGTTGGGAT

MCox2-PR1: FAM-CGTAGCTTCAGTATCATTGGTGCCCTATGGT-TAMRA

95 C, 9 min

95 C 30 sec, 62 C 30 sec for 55 cycles

(We thank W. Switzer for this protocol)
