# Supplementary material for: Multiple Sources of Contamination in Samples from Patients Reported to Have XMRV Infection
Source: PLoS One. 2012 Feb 20;7(2):e30889. doi: 10.1371/journal.pone.0030889 (PMC3282701; doi:10.1371/journal.pone.0030889)
Supplement: Appendix S4 — IAP detection protocol and primer sequences. (DOCX) [file pone.0030889.s004.docx]

**Appendix S4**

*IAP protocol and primer sequences*

IAP-F ATAATCTGCGCATGAGCCAAGG

IAP-R AGGAAGAACACCACAGACCAGA

45 cycles PCR:

95 degrees for 30 sec

60 degrees for 30 sec

72 degrees for 30 sec

(We thank O. Cingöz for this protocol)
